# Supplementary material for: Split Intein-Mediated Protein Ligation for detecting protein-protein interactions and their inhibition
Source: Nat Commun. 2020 May 15;11:2440. doi: 10.1038/s41467-020-16299-1 (PMC7229206; doi:10.1038/s41467-020-16299-1)
Supplement: Supplementary file 1 — Supplementary Information [file 41467_2020_16299_MOESM1_ESM.pdf]

Supplementary Information

Split Intein-Mediated Protein Ligation, a Method for Detecting Protein-Protein Interactions and Their Inhibition

Yao et al.

Supplementary Table 1. Different constructs of FRB and FKBP1A and their products used in Fig. 2-3

Name	Description	MW (kDa)	Protein sequence
FRB (IN)	FRB-HAx2-V5-EN-IN	29.5	MRVAILWHEMWHEGLEEASRLYFGERNVKGMFEVLEPLHAMMERGPQTLKETSFNQAYGR<br>DLMEAQEWCRKYMKSGVKDLTQAWDLYYHVFRRISYPAFLYKVVDSRGGGGSGGGGSYPY<br>DVPDYAGGSYPYDVPDYAGGGSGKPIPNPLLGLDSTTRSGYCLDLKTQVQTPQGMKEISN<br>IQVGDLVLSNTGYNEVLNVFPKSKKKSYKITLEDGKEIICSEEHLFPTQTGEMNISGGLK<br>EGMCLYVKEMMLKKILKIEEL
FRB (NIN)	HAx2-V5-EN-IN-FRB	30.7	MYPYDVPDYAGGSYPYDVPDYAGGGSGKPIPNPLLGLDSTTRSGYCLDLKTQVQTPQGMK<br>EISNIQVGDLVLSNTGYNEVLNVFPKSKKKSYKITLEDGKEIICSEEHLFPTQTGEMNIS<br>GGLKEGMCLYVKEMMLKKILKIEELGGSGGGGSGGGGSKLTSLYKKAGLMRVAILWHEMW<br>HEGLEEASRLYFGERNVKGMFEVLEPLHAMMERGPQTLKETSFNQAYGRDLMEAQEWCRK<br>YMKSGNVKDLTQAWDLYYHVFRRISYPAFLYKVV
FKBP1A (IC)	IC-EC-FLAGx3-Myc-FKBP1A	22.4	MDERELIDIEVSGNHLFYANDILTHNSSSDVGTDYKDHDGDYKDHDIDYKDDDDKGGGGS<br>GGGGSEQKLISEEDLLTSLYKKAGSTMGVQVETISPGDGRTFPKRGQTCVVHYTGMLEDG<br>KKFDSSRDRNKPFKFMLGKQEVIRGWEEGVAQMSVGQRAKLTISPDYAYGATGHPGIIPP<br>HATLVFDVELLKLESDPAFLYKVV
FKBP1A (CIC)	FKBP1A-IC-EC-FLAGx3-Myc	21.8	MGVQVETISPGDGRTFPKRGQTCVVHYTGMLEDGKKFDSSRDRNKPFKFMLGKQEVIRGW<br>EEGVAQMSVGQRAKLTISPDYAYGATGHPGIIPPHATLVFDVELLKLESDPAFLYKVVDS<br>RGGGGSGGGGSGMDERELIDIEVSGNHLFYANDILTHNSSSDVGTDYKDHDGDYKDHDID<br>YKDDDDKGSEQKLISEEDL
FKBP1A (CIC-GFP)	FKBP1A-IC-EC-FLAGx3-Myc-GFP	49.0	MGVQVETISPGDGRTFPKRGQTCVVHYTGMLEDGKKFDSSRDRNKPFKFMLGKQEVIRGW<br>EEGVAQMSVGQRAKLTISPDYAYGATGHPGIIPPHATLVFDVELLKLESDPAFLYKVVDS<br>RGGGGSGGGGSGMDERELIDIEVSGNHLFYANDILTHNSSSDVGTDYKDHDGDYKDHDID<br>YKDDDDKGSEQKLISEEDLSRMVSKGEELFTGVVPILVELDGDVNGHKFSVSGEGEGDAT<br>YGKLTLKFICTTGKLPVPWPTLVTTLTYGVQCFSRYPDHMKQHDFFKSAMPEGYVQERTI<br>FFKDDGNYKTRAEVKFEGDTLVNRIELKGIDFKEDGNILGHKLEYNYNSHNVYIMADKQK<br>NGIKVNFKIRHNIEDGSVQLADHYQQNTPIGDGPVLLPDNHYLSTQSALSKDPNEKRDHM<br>VLLEFVTAAGITLGMDELYK
Product of FRB (IN) + FKBP1A (IC)	FRB-HAx2-V5-EN-EC-FLAGx3-Myc-FKBP1A	37.6	MRVAILWHEMWHEGLEEASRLYFGERNVKGMFEVLEPLHAMMERGPQTLKETSFNQAYGR<br>DLMEAQEWCRKYMKSGNVKDLTQAWDLYYHVFRRISYPAFLYKVVDSRGGGGSGGGGSYP<br>YDVPDYAGGSYPYDVPDYAGGGSGKPIPNPLLGLDSTTRSGYSSSDVGTDYKDHDGDYKD<br>HDIDYKDDDDKGGGGSGGGGSEQKLISEEDLLTSLYKKAGSTMGVQVETISPGDGRTFPK<br>RGQTCVVHYTGMLEDGKKFDSSRDRNKPFKFMLGKQEVIRGWEEGVAQMSVGQRAKLTIS<br>PDYAYGATGHPGIIPPHATLVFDVELLKLESDPAFLYKVV
Product of FRB (IN) + FKBP1A (CIC)	FRB-HAx2-V5-EN-EC-FLAGx3-Myc	20.9	MRVAILWHEMWHEGLEEASRLYFGERNVKGMFEVLEPLHAMMERGPQTLKETSFNQAYGR<br>DLMEAQEWCRKYMKSGNVKDLTQAWDLYYHVFRRISYPAFLYKVVDSRGGGGSGGGGSYP<br>YDVPDYAGGSYPYDVPDYAGGGSGKPIPNPLLGLDSTTRSGYSSSDVGTDYKDHDGDYKD<br>HDIDYKDDDDKGSEQKLISEEDL
Product of FRB (NIN) + FKBP1A (IC)	HAx2-V5-EN-EC-FLAGx3-Myc-FKBP1A	24.1	MYPYDVPDYAGGSYPYDVPDYAGGGSGKPIPNPLLGLDSTTRSGYSSSDVGTDYKDHDGD<br>YKDHDIDYKDDDDKGGGGSGGGGSEQKLISEEDLLTSLYKKAGSTMGVQVETISPGDGRT<br>FPKRGQTCVVHYTGMLEDGKKFDSSRDRNKPFKFMLGKQEVIRGWEEGVAQMSVGQRAKL<br>TISPDYAYGATGHPGIIPPHATLVFDVELLKLESDPAFLYKVV
Product of FRB (NIN) + FKBP1A (CIC-GFP)	HAx2-V5-EN-EC-FLAGx3-Myc-GFP	36.6	MYPYDVPDYAGGSYPYDVPDYAGGGSGKPIPNPLLGLDSTTRSGYSSSDVGTDYKDHDGD<br>YKDHDIDYKDDDDKGSEQKLISEEDLSRMVSKGEELFTGVVPILVELDGDVNGHKFSVSG<br>EGEGDATYGKLTLKFICTTGKLPVPWPTLVTTLTYGVQCFSRYPDHMKQHDFFKSAMPEG<br>YVQERTIFFKDDGNYKTRAEVKFEGDTLVNRIELKGIDFKEDGNILGHKLEYNYNSHNVY<br>IMADKQKNGIKVNFKIRHNIEDGSVQLADHYQQNTPIGDGPVLLPDNHYLSTQSALSKDP<br>NEKRDHMVLLEFVTAAGITLGMDELYK

Supplementary Table 2. Reference sets for evaluating SIMPL assay.
(a) Positive reference set (PRS)

bait		prey	
**name**	**subcellular localization**	**name**	**subcellular localization**
AKT1	cell membrane (associated), cytoplasm, nucleus	PDPK1	plasma membrane (peripheral), nucleus, cytosol
AKT1	cell membrane (associated), cytoplasm, nucleus	TCL1A	nucleus, ER
ARF1	Golgi apparatus (anchored), plasma membrane (anchored)	ARFIP2	plasma membrane, cytosol, Golgi apparatus
ATF3	nucleus	DDIT3	nucleus
B2M	extracellular	HLA-A	plasma membrane, ER, Golgi apparatus
B2M	extracellular	HLA-B	plasma membrane, ER, Golgi apparatus
B2M	extracellular	HLA-C	plasma membrane, ER, Golgi apparatus
BAD	mitochondrion (outer membrane), cytoplasm	BCL2L1	mitochondrion, cytoskeleton, nucleus, cytosol
BAK1	mitochondrion (outer membrane)	BCL2L1	mitochondrion, cytoskeleton, nucleus, cytosol
BDNF	extracellular	NTF4	extracellular
CASP2	cytosol, mitochondrion, nucleus	CRADD	nucleus, cytosol
CBLB	cytoplasm	GRB2	plasma membrane, cytoplasm, nucleus
CCND3	nucleus	CDK6	nucleus, cytoskeleton, cytosol
CD2	cell membrane	CD58	plasma membrane
CDK2	nucleus, cytoplasm, cytoskeleton, endosome	CKS1B	nucleus
CDKN1A	nucleus	CCNA1	nucleus
CDKN1B	nucleus, endosome	CCNA1	nucleus
CEBPG	nucleus	FOS	nucleus, ER, cytosol
CGA	extracellular	CGB5	exracellular
CRK	cytoplasm, nucleus, membrane (associated)	PDGFRB	plasma membrane, lysosome
CXCL1	extracellular	CXCR2	plasma membrane
DDIT3	nucleus	FOS	nucleus, ER, cytosol
DR1	nucleus	DRAP1	nucleus
ERBB3	plasma membrane	NRG1	membrane, extracellular
FABP5	nucleus, cytosol, membrane, extracellular	S100A7	extracellular, cytoplasm, nucleus
FEN1	nucleus	PCNA	nucleus
FGF1	extracellular, cytosol, nucleus	FGFR1	plasma membrane
GADD45A	nucleus	PCNA	nucleus
GRAP2	endosome, cytosol, nucleus	LAT	plasma membrane
GRB2	plasma membrane, cytoplasm, nucleus	LAT	plasma membrane
GRB2	plasma membrane, cytoplasm, nucleus	PTK2	plasma membrane (associated), cytoskeleton, nucleus, cytosol
GRB2	plasma membrane, cytoplasm, nucleus	VAV1	cytosol, plasma membrane
GTF2F1	nucleus	GTF2F2	nucleus
HBA2	cytosol	HBB	cytosol
HDAC1	nucleus	RB1	nucleus
HDAC1	nucleus	ZBTB16	nucleus
HIF1A	nucleus, cytosol	TP53	nucleus, mitochodrion, ER
IFIT1	cytoplasm	EIF3E	nucleus, cytosol
IGF2	extracellular	IGFBP4	extracellular
JUNB	nucleus	BATF	nucleus
LCP2	cytoplasm	GRAP2	endosome, cytosol, nucleus
LCP2	cytoplasm	NCK1	plasma membrane, ER, nucleus, cytosol
LCP2	cytoplasm	VAV1	cytosol, plasma membrane
LGALS3	extracellular, plasma membrane, nucleus, cytoplasm	LGALS3BP	extracellular, nucleus, plasma membrane
LMNA	nucleus	LMNB1	nucleus
LMNA	nucleus	RB1	nucleus
LSM3	nucleus	LSM2	nucleus
MAD2L1	nucleus, cytoskeleton, kinetochore	MAD1L1	cytoskeleton, nucleus, kinetochore
MAFG	nucleus	NFE2L1	ER, nucleus
MAPK7	nucleus, cytoplasm	MAP2K5	nucleus, cytoplasm
MCM2	nucleus	MCM3	nucleus
MCM2	nucleus	MCM5	nucleus, cytosol
NCBP1	nucleus	NCBP2	nucleus
NF2	plasma membrane (associated), nucleus	HGS	endosome, cytoplasm
NR3C1	mitochondrion, cytosol, nucleus, cytoskeleton	HSP90AA1	plasma membrane, cytoplasm, nucleus
NR3C1	mitochondrion, cytosol, nucleus, cytoskeleton	RELA	nucleus, cytoplasm
ORC2	nucleus	MCM10	nucleus
ORC2	nucleus	ORC4	nucleus
PDE4D	plasma membrane (associated), cytoskeleton	RACK1	plasma membrane, nucleus, mitochondrion, cytosol
PDGFRB	plasma membrane, lysosome	PTPN11	nucleus, cytoplasm
PEX14	peroxisome	PEX19	peroxisome
PEX19	peroxisome	PEX11B	peroxisome
PEX19	peroxisome	PEX16	peroxisome
PEX19	peroxisome	PEX3	peroxisome
PPP3CA	nucleus, cytosol, plasma membrane (peripheral)	PPP3R1	cytosol, plasma membrane (anchored), nucleus
PRKAR2A	plasma membrane, cytoplasm, nucleus	EZR	plasma membrane (peripheral), cytoskeleton, nucleus, cytosol, endosome
PSMD4	cytosol, nucleus	RAD23A	nucleus, cytosol
PTK2	plasma membrane (associated), cytoskeleton, nucleus, cytosol	SRC	plasma membrane, cytoskeleton, cytoplasm, nucleus, mitochondrion
PTPN11	nucleus, cytoplasm	FRS2	plasma membrane, cytosol, endosome
RAC1	plasma membrane (anchored), cytoplasm	ARFIP2	plasma membrane, cytosol, Golgi apparatus
RAF1	cytosol, plasma membrane, nucleus	RAP1A	plasma membrane (anchored), endosome
RCC1	nucleus	RAN	nucleus, cytosol
RET	plasma membrane, endosome	FRS2	plasma membrane, cytosol, endosome
RHOA	cytoskeleton, plasma membrane (anchored)	ARHGAP1	cytoplasm
RIPK2	cytoplasm	NOD1	plasma membrane, cytoplasm
RPA2	nucleus	RPA3	nucleus
S100A1	nucleus, cytoplasm	S100B	nucleus, cytoplasm
S100A6	plasma membrane (peripheral), nucleus, cytoplasm	S100B	nucleus, cytoplasm
SKP1	cytoplasm, nucleus	BTRC	nucleus, cytosol
SKP1	cytoplasm, nucleus	SKP2	nucleus, cytoplasm
SMAD1	nucleus, cytoplasm	SMAD4	nucleus, cytoplasm
SMAD3	nucleus, cytoplasm	SMAD4	nucleus, cytoplasm
SMAD4	nucleus, cytoplasm	DCP1A	nucleus
TNFSF10	extracellular, plasma membrane	TNFRSF10B	plasma membrane
TP53	nucleus, mitochodrion, ER	UBE2I	nucleus, cytosol
XIAP	nucleus, cytoplasm	CASP3	cytoplasm, nucleus
XIAP	nucleus, cytoplasm	CASP7	cytoplasm, nucleus
XIAP	nucleus, cytoplasm	CASP9	cytosol, mitochondrion

- Continued

- Continuing

(b) Random reference set (RRS)

bait		prey	
**name**	**subcellular localization**	**name**	**subcellular localization**
AKT1	cell membrane (associated), cytoplasm, nucleus	PEX19	peroxisome
ARF1	Golgi apparatus (anchored), plasma membrane (anchored)	SKP2	nucleus, cytoplasm
ATF3	nucleus	LMNB1	nucleus
B2M	extracellular	PEX16	peroxisome
B2M	extracellular	RAD23A	nucleus, cytosol
B2M	extracellular	TP53	nucleus, mitochodrion, ER
BAD	mitochondrion (outer membrane), cytoplasm	DCP1A	nucleus
BAK1	mitochondrion (outer membrane)	RAP1A	plasma membrane (anchored), endosome
BDNF	extracellular	MAD1L1	cytoskeleton, nucleus, kinetochore
BDNF	extracellular	MCM5	nucleus, cytosol
CASP2	cytosol, mitochondrion, nucleus	VAV1	cytosol, plasma membrane
CBLB	cytoplasm	BCL2L1	mitochondrion, cytoskeleton, nucleus, cytosol
CD2	cell membrane	GTF2F2	nucleus
CD2	cell membrane	HGS	endosome, cytoplasm
CDK2	nucleus, cytoplasm, cytoskeleton, endosome	DDIT3	nucleus
CDKN1A	nucleus	PEX11B	peroxisome
CDKN1B	nucleus, endosome	DRAP1	nucleus
CEBPG	nucleus	ARHGAP1	cytoplasm
CEBPG	nucleus	CASP9	cytosol, mitochondrion
CGA	extracellular	CD58	plasma membrane
CGA	extracellular	MCM3	nucleus
CGA	extracellular	NCBP2	nucleus
CRK	cytoplasm, nucleus, membrane (associated)	UBE2I	nucleus, cytosol
CXCL1	extracellular	MAP2K5	nucleus, cytoplasm
CXCL1	extracellular	ORC4	nucleus
CXCL1	extracellular	SMAD4	nucleus, cytoplasm
DDIT3	nucleus	NRG1	membrane, extracellular
DR1	nucleus	NTF4	extracellular
ERBB3	plasma membrane	DDIT3	nucleus
ERBB3	plasma membrane	PCNA	nucleus
FABP5	nucleus, cytosol, membrane, extracellular	PTK2	plasma membrane (associated), cytoskeleton, nucleus, cytosol
FEN1	nucleus	GRAP2	endosome, cytosol, nucleus
FGF1	extracellular, cytosol, nucleus	NCK1	plasma membrane, ER, nucleus, cytosol
FGF1	extracellular, cytosol, nucleus	RB1	nucleus
GADD45A	nucleus	FRS2	plasma membrane, cytosol, endosome
GADD45A	nucleus	PDPK1	plasma membrane (peripheral), nucleus, cytosol
GRAP2	endosome, cytosol, nucleus	NFE2L1	ER, nucleus
GRB2	plasma membrane, cytoplasm, nucleus	PEX3	peroxisome
GTF2F1	nucleus	NOD1	plasma membrane, cytoplasm
HBA2	cytosol	CKS1B	nucleus
HDAC1	nucleus	LAT	plasma membrane
HDAC1	nucleus	PPP3R1	cytosol, plasma membrane (anchored), nucleus
HIF1A	nucleus, cytosol	ARFIP2	plasma membrane, cytosol, Golgi apparatus
IFIT1	cytoplasm	RELA	nucleus, cytoplasm
IGF2	extracellular	ARFIP2	plasma membrane, cytosol, Golgi apparatus
IGF2	extracellular	HSP90AA1	plasma membrane, cytoplasm, nucleus
IGF2	extracellular	RPA3	nucleus
JUNB	nucleus	PTPN11	nucleus, cytoplasm
LCP2	cytoplasm	CRADD	nucleus, cytosol
LGALS3	extracellular, plasma membrane, nucleus, cytoplasm	LSM2	nucleus
LMNA	nucleus	PEX3	peroxisome
LSM3	nucleus	FGFR1	plasma membrane
LSM3	nucleus	LGALS3BP	extracellular, nucleus, plasma membrane
MAD2L1	nucleus, cytoskeleton, kinetochore	NFE2L1	ER, nucleus
MAD2L1	nucleus, cytoskeleton, kinetochore	NTF4	extracellular
MAFG	nucleus	PEX11B	peroxisome
MAFG	nucleus	TNFRSF10B	plasma membrane
MAPK7	nucleus, cytoplasm	PEX16	peroxisome
MCM2	nucleus	S100B	nucleus, cytoplasm
NCBP1	nucleus	S100B	nucleus, cytoplasm
NF2	plasma membrane (associated), nucleus	FOS	nucleus, ER, cytosol
NR3C1	mitochondrion, cytosol, nucleus, cytoskeleton	RAD23A	nucleus, cytosol
ORC2	nucleus	HBB	cytosol
ORC2	nucleus	UBE2I	nucleus, cytosol
PDE4D	plasma membrane (associated), cytoskeleton	EZR	plasma membrane (peripheral), cytoskeleton, nucleus, cytosol, endosome
PDGFRB	plasma membrane, lysosome	RAN	nucleus, cytosol
PEX14	peroxisome	NRG1	membrane, extracellular
PEX19	peroxisome	BATF	nucleus
PPP3CA	nucleus, cytosol, plasma membrane (peripheral)	FOS	nucleus, ER, cytosol
PRKAR2A	plasma membrane, cytoplasm, nucleus	SRC	plasma membrane, cytoskeleton, cytoplasm, nucleus, mitochondrion
PSMD4	cytosol, nucleus	NOD1	plasma membrane, cytoplasm
PSMD4	cytosol, nucleus	TCL1A	nucleus, ER
RAC1	plasma membrane (anchored), cytoplasm	BTRC	nucleus, cytosol
RCC1	nucleus	CASP7	cytoplasm, nucleus
RET	plasma membrane, endosome	NCBP2	nucleus
RHOA	cytoskeleton, plasma membrane (anchored)	CCNA1	nucleus
RHOA	cytoskeleton, plasma membrane (anchored)	ZBTB16	nucleus
RIPK2	cytoplasm	IGFBP4	extracellular
RIPK2	cytoplasm	PDGFRB	plasma membrane, lysosome
RPA2	nucleus	S100A7	extracellular, cytoplasm, nucleus
S100A1	nucleus, cytoplasm	CASP3	cytoplasm, nucleus
S100A6	plasma membrane (peripheral), nucleus, cytoplasm	RAN	nucleus, cytosol
SKP1	cytoplasm, nucleus	CXCR2	plasma membrane
SMAD1	nucleus, cytoplasm	GRB2	plasma membrane, cytoplasm, nucleus
SMAD3	nucleus, cytoplasm	CXCR2	plasma membrane
SMAD4	nucleus, cytoplasm	LAT	plasma membrane
TNFSF10	extracellular, plasma membrane	CDK6	nucleus, cytoskeleton, cytosol
XIAP	nucleus, cytoplasm	ARFIP2	plasma membrane, cytosol, Golgi apparatus

Supplementary Table 3. Detection of reference sets with SIMPL.

(a) Detection with SIMPL in bait-IN/IC-prey format

bait	prey	reference set	RLU_mean	RLU_sd	SIMPL result
RPA2	RPA3	PRS	3.4432	0.3631	positive
DR1	DRAP1	PRS	2.8255	0.4781	positive
CCND3	CDK6	PRS	2.6886	0.2360	positive
JUNB	BATF	PRS	2.6670	0.3886	positive
LSM3	LSM2	PRS	2.5840	0.3028	positive
GTF2F1	GTF2F2	PRS	2.1478	0.3250	positive
BAK1	BCL2L1	PRS	2.0739	0.2237	positive
PEX14	PEX19	PRS	2.0669	0.1983	positive
CBLB	GRB2	PRS	1.9893	0.6659	positive
HBA2	HBB	PRS	1.8942	0.0060	positive
PEX19	PEX3	PRS	1.8937	0.0574	positive
BAD	BCL2L1	PRS	1.7771	0.2071	positive
PPP3CA	PPP3R1	PRS	1.7320	0.2225	positive
CDK2	CKS1B	PRS	1.6499	0.1770	positive
ERBB3	NRG1	PRS	1.5434	0.6700	positive
FEN1	PCNA	PRS	1.2627	0.1536	positive
ATF3	DDIT3	PRS	1.2622	0.1849	positive
LCP2	GRAP2	PRS	1.1838	0.1851	positive
S100A1	S100B	PRS	1.1498	0.1606	positive
LMNA	LMNB1	PRS	1.1180	0.1548	positive
NF2	HGS	PRS	1.0724	0.2408	positive
NCBP1	NCBP2	PRS	1.0597	0.1701	positive
PEX19	PEX16	PRS	1.0292	0.1177	positive
HIF1A	TP53	PRS	1.0262	0.1409	positive
RIPK2	NOD1	PRS	1.0134	0.1334	positive
SKP1A	BTRC	PRS	1.0033	0.0818	positive
CASP2	CRADD	PRS	0.9343	0.0988	positive
PEX19	PEX11B	PRS	0.8860	0.0670	positive
TNFSF10	TNFRSF10B	PRS	0.8622	0.1170	positive
GADD45A	FRS2	RRS	0.8477	0.1434	positive
XIAP	CASP9	PRS	0.8388	0.0395	positive
RET	FRS2	PRS	0.7796	0.1145	positive
CASP2	VAV1	RRS	0.7738	0.4035	positive
FABP5	S100A7	PRS	0.7703	0.0242	positive
CEBPG	FOS	PRS	0.7644	0.3913	positive
MAFG	NFE2L1	PRS	0.7280	0.2500	positive
CD2	CD58	PRS	0.7039	0.0441	positive
RIPK2	PDGFRB	RRS	0.7012	0.0385	positive
HDAC1	ZBTB16	PRS	0.6820	0.4423	positive
RET	NCBP2	RRS	0.6734	0.3991	positive
IFIT1	EIF3E	PRS	0.6638	0.3544	positive
LMNA	RB1	PRS	0.6623	0.2735	positive
IGF2	IGFBP4	PRS	0.6385	0.1957	positive
NR3C1	HSP90AA1	PRS	0.6146	0.0308	positive
RCC1	RAN	PRS	0.6139	0.1312	positive
RIPK2	IGFBP4	RRS	0.6059	0.0301	negative
PDE4D	RACK1	PRS	0.5972	0.0294	negative
HDAC1	RB1	PRS	0.5959	0.0286	negative
PEX14	NRG1	RRS	0.5888	0.0438	negative
S100A6	S100B	PRS	0.5874	0.0182	negative
GADD45A	PDPK1	RRS	0.5841	0.0095	negative
MAPK7	MAP2K5	PRS	0.5642	0.0233	negative
PSMD4	RAD23A	PRS	0.5577	0.0557	negative
IGF2	RPA3	RRS	0.5443	0.0322	negative
CDKN1A	CCNA1	PRS	0.5356	0.1474	negative
PTK2	SRC	PRS	0.5319	0.0367	negative
RAF1	RAP1A	PRS	0.5305	0.0936	negative
NR3C1	RELA	PRS	0.5280	0.1268	negative
LCP2	NCK1	PRS	0.4820	0.0316	negative
BAD	DCP1A	RRS	0.4796	0.0683	negative
NF2	FOS	RRS	0.4749	0.0968	negative
SMAD4	DCP1A	PRS	0.4734	0.0803	negative
S100A1	CASP3	RRS	0.4714	0.0458	negative
MCM2	MCM3	PRS	0.4644	0.0526	negative
RAC1	ARFIP2	PRS	0.4452	0.0384	negative
BAK1	RAP1A	RRS	0.4413	0.1913	negative
DDIT3	FOS	PRS	0.4361	0.0525	negative
FGF1	NCK1	RRS	0.4314	0.0122	negative
MAFG	PEX11B	RRS	0.4226	0.0877	negative
CEBPG	CASP9	RRS	0.4163	0.1929	negative
PTPN11	FRS2	PRS	0.4053	0.1141	negative
ARF1	ARFIP2	PRS	0.4037	0.0272	negative
IFIT1	RELA	RRS	0.3984	0.0852	negative
MAFG	TNFRSF10B	RRS	0.3964	0.0726	negative
HIF1A	ARFIP2	RRS	0.3917	0.0704	negative
ORC2	MCM10	PRS	0.3911	0.0303	negative
ATF3	LMNB1	RRS	0.3878	0.0598	negative
BDNF	NTF4	PRS	0.3704	0.0853	negative
CRK	UBE2I	RRS	0.3665	0.0331	negative
CDKN1B	CCNA1	PRS	0.3659	0.1165	negative
IGF2	HSP90AA1	RRS	0.3610	0.1050	negative
MAD2L1	MAD1L1	PRS	0.3560	0.0458	negative
AKT1	PDPK1	PRS	0.3493	0.0243	negative
ORC2	ORC4	PRS	0.3385	0.0656	negative
MCM2	S100B	RRS	0.3360	0.0200	negative
RHOA	ARHGAP1	PRS	0.3216	0.0633	negative
IGF2	ARFIP2	RRS	0.3180	0.2286	negative
NCBP1	S100B	RRS	0.3175	0.1453	negative
AKT1	TCL1A	PRS	0.3071	0.0383	negative
BDNF	MAD1L1	RRS	0.3025	0.1799	negative
JUNB	PTPN11	RRS	0.2965	0.0640	negative
ERBB3	DDIT3	RRS	0.2921	0.0671	negative
PDGFRB	PTPN11	PRS	0.2892	0.0103	negative
TNFSF10	CDK6	RRS	0.2847	0.0237	negative
FGF1	RB1	RRS	0.2767	0.0291	negative
XIAP	ARFIP2	RRS	0.2730	0.0261	negative
SMAD3	SMAD4	PRS	0.2678	0.0461	negative
BDNF	MCM5	RRS	0.2600	0.0444	negative
PSMD4	TCL1A	RRS	0.2458	0.0368	negative
CGA	CGB5	PRS	0.2458	0.0653	negative
XIAP	CASP3	PRS	0.2444	0.0266	negative
CXCL1	CXCR2	PRS	0.2441	0.0244	negative
HDAC1	PPP3R1	RRS	0.2428	0.1576	negative
CD2	HGS	RRS	0.2394	0.0189	negative
CBLB	BCL2L1	RRS	0.2388	0.0662	negative
XIAP	CASP7	PRS	0.2274	0.0121	negative
FEN1	GRAP2	RRS	0.2249	0.0246	negative
PEX19	BATF	RRS	0.2231	0.0156	negative
AKT1	PEX19	RRS	0.2170	0.1564	negative
HDAC1	LAT	RRS	0.2144	0.0078	negative
SMAD4	LAT	RRS	0.2118	0.0374	negative
TP53	UBE2I	PRS	0.2117	0.0324	negative
HBA2	CKS1B	RRS	0.2111	0.0309	negative
RHOA	ZBTB16	RRS	0.2108	0.0398	negative
CGA	CD58	RRS	0.2106	0.0212	negative
GRAP2	LAT	PRS	0.2084	0.0407	negative
GRB2	PTK2	PRS	0.2078	0.0279	negative
MCM2	MCM5	PRS	0.2022	0.0462	negative
NR3C1	RAD23A	RRS	0.1958	0.1008	negative
SMAD1	SMAD4	PRS	0.1937	0.0254	negative
ARF1	SKP2	RRS	0.1903	0.0234	negative
LCP2	VAV1	PRS	0.1881	0.0068	negative
LSM3	LGALS3BP	RRS	0.1875	0.0665	negative
B2M	HLA-C	PRS	0.1875	0.0347	negative
FABP5	PTK2	RRS	0.1837	0.0038	negative
LGALS3	LSM2	RRS	0.1794	0.0490	negative
CDK2	DDIT3	RRS	0.1786	0.0530	negative
PSMD4	NOD1	RRS	0.1762	0.0201	negative
LSM3	FGFR1	RRS	0.1749	0.0246	negative
PDGFRB	RAN	RRS	0.1711	0.0369	negative
ORC2	HBB	RRS	0.1695	0.0481	negative
RAC1	BTRC	RRS	0.1679	0.0335	negative
ORC2	UBE2I	RRS	0.1663	0.0149	negative
DDIT3	NRG1	RRS	0.1649	0.0664	negative
MAD2L1	NTF4	RRS	0.1647	0.0587	negative
CDKN1A	PEX11B	RRS	0.1630	0.1186	negative
GRB2	LAT	PRS	0.1585	0.0421	negative
RCC1	CASP7	RRS	0.1530	0.0145	negative
RHOA	CCNA1	RRS	0.1528	0.1188	negative
LMNA	PEX3	RRS	0.1484	0.0408	negative
S100A6	RAN	RRS	0.1435	0.0207	negative
LGALS3	LGALS3BP	PRS	0.1411	0.0338	negative
GADD45A	PCNA	PRS	0.1400	0.0118	negative
PPP3CA	FOS	RRS	0.1371	0.0615	negative
PDE4D	EZR	RRS	0.1367	0.0274	negative
B2M	HLA-A	PRS	0.1366	0.0339	negative
CDKN1B	DRAP1	RRS	0.1353	0.0227	negative
RPA2	S100A7	RRS	0.1315	0.0147	negative
B2M	HLA-B	PRS	0.1279	0.0164	negative
SKP1A	SKP2	PRS	0.1250	0.0104	negative
ERBB3	PCNA	RRS	0.1231	0.0572	negative
LCP2	CRADD	RRS	0.1229	0.0160	negative
SMAD3	CXCR2	RRS	0.1226	0.0229	negative
GTF2F1	NOD1	RRS	0.1222	0.0076	negative
SMAD1	GRB2	RRS	0.1194	0.0140	negative
GRB2	VAV1	PRS	0.1189	0.0135	negative
FGF1	FGFR1	PRS	0.1174	0.0158	negative
MAPK7	PEX16	RRS	0.1127	0.0193	negative
CRK	PDGFRB	PRS	0.1125	0.0247	negative
PRKAR2A	SRC	RRS	0.1114	0.0122	negative
SKP1A	CXCR2	RRS	0.1059	0.0220	negative
PRKAR2A	EZR	PRS	0.1054	0.0162	negative
CD2	GTF2F2	RRS	0.1015	0.0233	negative
CXCL1	SMAD4	RRS	0.0943	0.0366	negative
CXCL1	MAP2K5	RRS	0.0939	0.0254	negative
CXCL1	ORC4	RRS	0.0832	0.0224	negative
CGA	MCM3	RRS	0.0812	0.0197	negative
DR1	NTF4	RRS	0.0810	0.0256	negative
GRAP2	NFE2L1	RRS	0.0807	0.0225	negative
CGA	NCBP2	RRS	0.0703	0.0116	negative
B2M	PEX16	RRS	0.0630	0.0070	negative
B2M	TP53	RRS	0.0561	0.0060	negative
CEBPG	ARHGAP1	RRS	0.0555	0.0444	negative
MAD2L1	NFE2L1	RRS	0.0465	0.0124	negative
GRB2	PEX3	RRS	0.0408	0.0085	negative
B2M	RAD23A	RRS	0.0389	0.0088	negative

(b) Detection with SIMPL in bait-IN/prey-CIC format

bait	prey	reference set	RLU_mean	RLU_sd	SIMPL result
DR1	DRAP1	PRS	3.9699	0.4737	positive
GTF2F1	GTF2F2	PRS	3.2408	0.3796	positive
LSM3	LSM2	PRS	3.0343	0.2997	positive
CCND3	CDK6	PRS	2.7241	0.4935	positive
RPA2	RPA3	PRS	2.7224	0.2376	positive
CBLB	GRB2	PRS	2.6123	0.6210	positive
MAD2L1	MAD1L1	PRS	2.5199	0.2689	positive
CASP2	CRADD	PRS	2.4486	1.2686	positive
PEX14	PEX19	PRS	2.1671	0.0056	positive
NCBP1	NCBP2	PRS	2.1468	0.1112	positive
JUNB	BATF	PRS	2.0388	0.1987	positive
CDK2	CKS1B	PRS	1.8825	0.1868	positive
LCP2	GRAP2	PRS	1.8606	0.2139	positive
BAD	BCL2L1	PRS	1.8522	0.2456	positive
BAK1	BCL2L1	PRS	1.7835	0.0973	positive
PEX19	PEX3	PRS	1.7696	0.1654	positive
MAFG	NFE2L1	PRS	1.7172	0.1158	positive
PPP3CA	PPP3R1	PRS	1.6785	0.1617	positive
ATF3	DDIT3	PRS	1.6376	0.1419	positive
GRB2	VAV1	PRS	1.3884	0.1383	positive
HBA2	HBB	PRS	1.3613	0.2704	positive
SKP1A	BTRC	PRS	1.2554	0.1391	positive
ERBB3	NRG1	PRS	1.2192	0.4106	positive
LMNA	RB1	PRS	1.1834	0.9960	positive
PEX19	PEX11B	PRS	1.1795	0.2065	positive
TNFSF10	TNFRSF10B	PRS	1.1777	0.0620	positive
RIPK2	NOD1	PRS	1.1730	0.1605	positive
PEX19	PEX16	PRS	1.0916	0.1442	positive
RET	NCBP2	RRS	1.0605	0.9735	positive
HIF1A	TP53	PRS	1.0160	0.0937	positive
CD2	CD58	PRS	0.9721	0.0868	positive
MAPK7	MAP2K5	PRS	0.9482	0.0931	positive
IGF2	IGFBP4	PRS	0.9430	0.1935	positive
MCM2	MCM3	PRS	0.9380	0.0613	positive
PSMD4	RAD23A	PRS	0.9194	0.1317	positive
RIPK2	PDGFRB	RRS	0.8813	0.1399	positive
LMNA	LMNB1	PRS	0.8612	0.1227	positive
PSMD4	NOD1	RRS	0.8452	0.3046	positive
NF2	HGS	PRS	0.8360	0.0436	positive
S100A1	S100B	PRS	0.8221	0.0467	positive
XIAP	CASP9	PRS	0.8110	0.0418	positive
LCP2	VAV1	PRS	0.7895	0.1110	positive
CEBPG	FOS	PRS	0.7883	0.3406	positive
RCC1	RAN	PRS	0.7479	0.0959	positive
GRB2	PTK2	PRS	0.7407	0.0235	positive
CASP2	VAV1	RRS	0.6955	0.1239	positive
RHOA	ZBTB16	RRS	0.6910	0.1301	positive
CDKN1B	CCNA1	PRS	0.6855	0.0124	positive
IFIT1	EIF3E	PRS	0.6758	0.2972	positive
RAC1	ARFIP2	PRS	0.6722	0.1864	positive
LCP2	NCK1	PRS	0.6615	0.0610	positive
NR3C1	HSP90AA1	PRS	0.6525	0.1639	positive
DDIT3	FOS	PRS	0.6418	0.0908	positive
PDE4D	RACK1	PRS	0.6388	0.0747	positive
NR3C1	RELA	PRS	0.6301	0.0899	positive
BDNF	NTF4	PRS	0.6288	0.0457	positive
RET	FRS2	PRS	0.6082	0.1119	positive
PTK2/FAK	SRC	PRS	0.5989	0.0745	positive
RAF1	RAP1A	PRS	0.5779	0.2303	positive
BAK1	RAP1A	RRS	0.5644	0.0331	negative
RIPK2	IGFBP4	RRS	0.5551	0.0974	negative
HDAC1	RB1	PRS	0.5398	0.1375	negative
PDGFRB	PTPN11	PRS	0.5394	0.1505	negative
IGF2	RPA3	RRS	0.5245	0.0746	negative
CDKN1A	CCNA1	PRS	0.5163	0.0793	negative
BAD	DCP1A	RRS	0.4988	0.0534	negative
HDAC1	ZBTB16	PRS	0.4919	0.0272	negative
ARF1	ARFIP2	PRS	0.4912	0.0746	negative
AKT1	PDPK1	PRS	0.4862	0.0688	negative
RHOA	ARHGAP1	PRS	0.4860	0.0238	negative
CEBPG	CASP9	RRS	0.4815	0.1080	negative
NF2	FOS	RRS	0.4805	0.1887	negative
PTPN11	FRS2	PRS	0.4742	0.2063	negative
MAFG	TNFRSF10B	RRS	0.4586	0.0308	negative
XIAP	CASP7	PRS	0.4519	0.0327	negative
XIAP	ARFIP2	RRS	0.4394	0.0555	negative
CGA	CGB5	PRS	0.4354	0.0710	negative
ORC2	MCM10	PRS	0.4298	0.0029	negative
IGF2	HSP90AA1	RRS	0.4038	0.1934	negative
SMAD3	SMAD4	PRS	0.3987	0.1559	negative
SMAD4	DCP1A	PRS	0.3982	0.0498	negative
B2M	HLA-C	PRS	0.3857	0.0356	negative
SKP1A	SKP2	PRS	0.3757	0.0473	negative
FGF1	NCK1	RRS	0.3739	0.0573	negative
RHOA	CCNA1	RRS	0.3597	0.2956	negative
MCM2	MCM5	PRS	0.3498	0.0162	negative
FEN1	PCNA	PRS	0.3494	0.0204	negative
JUNB	PTPN11	RRS	0.3453	0.0038	negative
FEN1	GRAP2	RRS	0.3419	0.0204	negative
MAFG	PEX11B	RRS	0.3415	0.0753	negative
FGF1	FGFR1	PRS	0.3343	0.1035	negative
IFIT1	RELA	RRS	0.3275	0.0894	negative
FGF1	RB1	RRS	0.3243	0.0702	negative
HIF1A	ARFIP2	RRS	0.3242	0.1547	negative
TNFSF10	CDK6	RRS	0.3161	0.0470	negative
XIAP	CASP3	PRS	0.3157	0.1010	negative
PEX14	NRG1	RRS	0.3098	0.0214	negative
B2M	HLA-B	PRS	0.3055	0.0047	negative
ARF1	SKP2	RRS	0.3012	0.0463	negative
CD2	GTF2F2	RRS	0.2986	0.0331	negative
ORC2	ORC4	PRS	0.2975	0.0677	negative
B2M	HLA-A	PRS	0.2883	0.0052	negative
ERBB3	DDIT3	RRS	0.2797	0.0806	negative
CDK2	DDIT3	RRS	0.2752	0.0283	negative
CBLB	BCL2L1	RRS	0.2722	0.0937	negative
LSM3	FGFR1	RRS	0.2720	0.0249	negative
CXCL1	MAP2K5	RRS	0.2696	0.0394	negative
IGF2	ARFIP2	RRS	0.2656	0.2095	negative
GADD45A	PDPK1	RRS	0.2649	0.0321	negative
CRK	PDGFRB	PRS	0.2606	0.0128	negative
RAC1	BTRC	RRS	0.2564	0.0248	negative
CD2	HGS	RRS	0.2532	0.0240	negative
LGALS3	LGALS3BP	PRS	0.2476	0.0370	negative
HDAC1	PPP3R1	RRS	0.2470	0.1233	negative
PPP3CA	FOS	RRS	0.2452	0.0635	negative
MAPK7	PEX16	RRS	0.2305	0.1169	negative
CDKN1B	DRAP1	RRS	0.2288	0.0131	negative
ORC2	HBB	RRS	0.2271	0.0370	negative
LMNA	PEX3	RRS	0.2245	0.0857	negative
GRB2	LAT	PRS	0.2228	0.0406	negative
HDAC1	LAT	RRS	0.2217	0.0425	negative
BDNF	MCM5	RRS	0.2203	0.0370	negative
AKT1	PEX19	RRS	0.2185	0.1698	negative
S100A6	S100B	PRS	0.2174	0.0799	negative
NCBP1	S100B	RRS	0.2169	0.0354	negative
SMAD4	LAT	RRS	0.2147	0.0334	negative
GADD45A	FRS2	RRS	0.2103	0.0296	negative
GRAP2	LAT	PRS	0.1945	0.0178	negative
TP53	UBE2I	PRS	0.1942	0.0436	negative
LCP2	CRADD	RRS	0.1931	0.0522	negative
BDNF	MAD1L1	RRS	0.1904	0.0314	negative
PRKAR2A	SRC	RRS	0.1857	0.0322	negative
NR3C1	RAD23A	RRS	0.1833	0.1110	negative
SMAD1	SMAD4	PRS	0.1817	0.0359	negative
CXCL1	CXCR2	PRS	0.1776	0.0135	negative
FABP5	PTK2	RRS	0.1738	0.0146	negative
HBA2	CKS1B	RRS	0.1709	0.0175	negative
LGALS3	LSM2	RRS	0.1624	0.0096	negative
S100A1	CASP3	RRS	0.1580	0.0237	negative
PEX19	BATF	RRS	0.1536	0.0259	negative
CRK	UBE2I	RRS	0.1527	0.0071	negative
SMAD1	GRB2	RRS	0.1512	0.0226	negative
SMAD3	CXCR2	RRS	0.1435	0.0096	negative
PDGFRB	RAN	RRS	0.1430	0.0191	negative
PDE4D	EZR	RRS	0.1367	0.0023	negative
ATF3	LMNB1	RRS	0.1344	0.0171	negative
FABP5	S100A7	PRS	0.1337	0.0375	negative
MAD2L1	NTF4	RRS	0.1332	0.0200	negative
GTF2F1	NOD1	RRS	0.1326	0.0052	negative
GRAP2	NFE2L1	RRS	0.1311	0.0270	negative
CGA	CD58	RRS	0.1307	0.0079	negative
LSM3	LGALS3BP	RRS	0.1293	0.0034	negative
RCC1	CASP7	RRS	0.1247	0.0522	negative
PRKAR2A	EZR	PRS	0.1234	0.0382	negative
ORC2	UBE2I	RRS	0.1188	0.0276	negative
CDKN1A	PEX11B	RRS	0.1133	0.0686	negative
AKT1	TCL1A	PRS	0.1105	0.0130	negative
PSMD4	TCL1A	RRS	0.1084	0.0091	negative
SKP1A	CXCR2	RRS	0.1068	0.0148	negative
S100A6	RAN	RRS	0.1055	0.0183	negative
CXCL1	ORC4	RRS	0.1032	0.0256	negative
MCM2	S100B	RRS	0.1016	0.0121	negative
CXCL1	SMAD4	RRS	0.1012	0.0196	negative
DDIT3	NRG1	RRS	0.0963	0.0350	negative
MAD2L1	NFE2L1	RRS	0.0819	0.0024	negative
ERBB3	PCNA	RRS	0.0812	0.0272	negative
CGA	MCM3	RRS	0.0738	0.0186	negative
GADD45A	PCNA	PRS	0.0617	0.0027	negative
RPA2	S100A7	RRS	0.0606	0.0032	negative
GRB2	PEX3	RRS	0.0588	0.0261	negative
CEBPG	ARHGAP1	RRS	0.0585	0.0258	negative
DR1	NTF4	RRS	0.0529	0.0074	negative
B2M	PEX16	RRS	0.0408	0.0101	negative
CGA	NCBP2	RRS	0.0363	0.0066	negative
B2M	TP53	RRS	0.0329	0.0073	negative
B2M	RAD23A	RRS	0.0155	0.0030	negative

Supplementary Table 4. Mitochondrial PPIs tested by SIMPL.

Bait				Prey				Function	Reference
**Name**	**Alias**	**Transit signal**	**Localization**	**Name**	**Alias**	**Transit signal**	**Localization**		
SDHA	SDH1, SDHF	N-terminal	Matrix protein peripheral to inner membrane	SDHB	SDH2	N terminal	Matrix protein peripheral to inner membrane	complex II	Sun et al, Cell (2005) 121, 1043-1057
ETFA	alpha-ETF	N-terminal	Matrix	ETFB	beta-ETF	unknown	Matrix	ETF complex, OXPHOS complexes	Toogood et al, J Biol Chem (2004) 279, 32904-32912
ETFA	alpha-ETF	N-terminal	Matrix	ETFRF1	LYRM5	unknown	Matrix	ETF complex, OXPHOS complexes	Floyd et al, Mol Cell (2016) 63, 621-632
TIMM10	TIM10	middle	Intermembrane space	TIMM9	TIM9	middle	Intermembrane space	TIM9/10 complex.	Webb et al, Mol Cell (2006) 21, 123-133
CHCHD6	MIC25	unknown	Lipid-anchored to inner membrane in the intermembrane space.	CHCHD3	MIC19	unknown	Lipid-anchored to inner membrane in the intermembrane space.	MICOS complex	Kozjak-Pavlovic, Cell Tissue Res (2017) 367: 83-93
UQCRC2	QCR2, UQCR2	N-terminal	Matrix side peripheral protein to inner membrane	UQCRC2	QCR2, UQCR2	N-terminal	Matrix side peripheral protein to inner membrane	complex III	Guo et al, Cell (2017) 170, 1247-1257
TIMM50	TIM50, TIM50L	N-terminal	Transmembrane protein at the inner mitochondrial membrane, exposing the C-terminus to the intermembrane space with interacting with TIMM23 N-terminal domain	TIMM23	TIM23	unknown	Inner membrane multiple transmembrane protein with N-terminus in the intermembrane space	TIM23 complex	Geissler et al, Cell (2002) 111, 507-518; Yamamoto et al, Cell 111 (2002), 519-528; Demishtein-Zohary et al, Cell Tissue Res (2017) 367, 33-41
ATP5MC1	ATP5G1, ATP5A, ATP5G	N-terminal	Multipass transmembrane protein in the inner membrane with both N- and C-termini in the intermembrane space	ATP5MC1	ATP5G1, ATP5A, ATP5G	N-terminal	Multipass transmembrane protein in the inner membrane with both N- and C-termini in the intermembrane space	Fo complex of ATPase	Rastogi et al, Nature (1999) 402, 263-268
NDUFV1	UQOR1, CI51KD	N-terminal	Matrix protein peripheral to inner membrane	NDUFV3	CI9KD	N-terminal	Matrix protein peripheral to inner membrane	N module of complex I	Guo et al, Cell (2017) 170, 1247-1257
PDHA1	PHE1A, PDHA, PDHAD	N-terminal	Matrix	PDHB	PHE1B, PDHE1B, PDHBD	N-terminal	Matrix	Pyruvate dehydrogenase complex	Ciszak et al, J Biol Chem (2003) 278, 21240-21246

Supplementary Table 5. C. elegans reference set studied by SIMPL

#	Reference Set	Bait Protein (IN-V5/HA) CDS	Bait Protein (IN-V5/HA) Protein	Prey Protein (IC/CIC-FLAG) CDS	Prey Protein (IC/CIC-FLAG) Protein	Configuration IN/IC	Configuration IN/CIC	ELISA Value Replicate 1 IN/IC	ELISA Value Replicate 1 IN/CIC	ELISA Value Replicate 2 IN/IC	ELISA Value Replicate 2 IN/CIC	PPI Description/Homologs	Source	Pubmed ID
1	Positive	T05H4.2	**FBXA-196**	F46A9.4	**SKR-2**	**Yes**	**Yes**	0.81	1.1	0.51	0.28	SKP1/F-Box Domain	CePRS	
2	Positive	Y45F10C.3	**FBXA-215**	F46A9.4	**SKR-2**	**Yes**	**Yes**	0.84	0.73	0.58	0.42	SKP1/F-Box Domain	CePRS	
3	Positive	ZK792.6	**LET-60**	AC7.2	**SOC-2**	**Yes**	**Yes**	1.2	0.79	0.59	0.70	SHOC2/HRAS	CePRS	
4	Positive	F53F10.5	**NPP-11**	K07F5.13	**NPP-1**	**Yes**	inj.	1.8	-	0.92	-	Nuclear pore complex components Nup54 and Nup62	LIT/EE	7531196
5	Positive	F58F6.4	**RFC-2**	F44B9.8	**F44B9.8**	**Yes**	inj.	0.66	-	0.88	-	RFC5/RFC2 Replication factor subunits	LIT/EE	15201901
6	Positive	T10E9.1	**T10E9.1**	F46A9.4	**SKR-2**	**Yes**	inj.	0.78	-	0.55	-	SKP1/F-Box Domain	CePRS	
7	Positive	T05G5.3	**CDK-1**	ZC168.4	**CYB-1**	inj.	**Yes**	-	1.1	-	0.80	Cyclin B2/Cyclin Dependent Kinase 1	LIT/EE	7575488
8	Positive	C06G3.10	**COGC-2**	Y51H7C.6	COGC-4	n.c.	n.c.	-	-	-	-	COG2/COG4 (component of oligomeric golgi complex 4)	LIT/EE	15047703
9	Positive	F10B5.6	**EMB-27**	B0511.9	**CDC-26**	inj.	inj.	-	-	-	-	Cdc26p and Cdc16p components of the anaphase promoting complex	LIT/EE	8895471
10	Positive	K08B4.1	**LAG-1**	C32A3.1	**SEL-8**	n.c.	n.c.	-	-	-	-	CSL (*C* BF-1, *S* u(H), *L* ag-1)/Mastermind	CePRS	
11	Positive	F58A3.1	**LDB-1**	F46C8.5	**CEH-14**	n.c.	n.c.	-	-	-	-	LDB2 (LIM domain binding 2)/LHX3 (LIM homeobox 3) and LHX4 (LIM homeobox 4)	CePRS	
12	Positive	M7.1	**LET-70**	F54G8.4	**NHL-1**	n.c.	n.c.	-	-	-	-	UBE2D2 (ubiquitin conjugating enzyme E2 D2)/TRIM2 (tripartite motif containing 2) and TRIM3 (tripartite motif containing 3)	CePRS	
13	Positive	F59E12.5	**NPL-4.2**	F19B6.2	**UFD-1**	n.c.	n.c.	-	-	-	-	NPLOC4 (NPL4 homolog, ubiquitin recognition factor)/UFD1 (ubiquitin recognition factor in ER associated degradation 1)	LIT/EE	10811609
14	Positive	F59A2.1	**NPP-9**	K01G5.4	**RAN-1**	n.c.	n.c.	-	-	-	-	RANBP1 (RAN binding protein 1)/RAN (RAN, member RAS oncogene family)	LIT/EE	7603572
15	Positive	F21C3.4	**RDE-2**	F21C3.4	**RDE-2**	n.c.	n.c.	-	-	-	-	RDE2 RNA interference protein	CePRS	
16	Positive	M03D4.1	**ZEN-4**	B0207.4	**AIR-2**	inj.	inj.	-	-	-	-	Aurora Kinase C/KIF23	CePRS	
17	Positive	Y37H2A.5	**FBXA-210**	F46A9.4	**SKR-2**	inj.	inj.	-	-	-	-	SKP1/F-Box Domain	CePRS	
18	Positive	Y113G7B.5	**FOG-2**	T23G11.3	**GLD-1**	inj.	inj.	-	-	-	-	QKI (QKI, KH domain containing RNA binding)/Germline protein	CePRS	
19	Positive	M7.1	**LET-70**	F16A11.1	**F16A11.1**	n.c.	n.c.	-	-	-	-	UBE2D2 (ubiquitin conjugating enzyme E2 D2)/RSPRY1 (ring finger and SPRY domain containing 1	CePRS	
20	Positive	M7.1	**LET-70**	C45G7.4	**C45G7.4**	n.c.	n.c.	-	-	-	-	UBE2D2 (ubiquitin conjugating enzyme E2 D2)/TRIM13	CePRS	
21	Positive	ZK1098.8	**MUT-7**	F21C3.4	**RDE-2**	n.c.	n.c.	-	-	-	-	EXD3 (exonuclease 3'-5' domain containing 3)/RDE2 involved in RNA interference	CePRS	
22	Positive	R06F6.5	**NPP-19**	Y37E3.15	**NPP-13**	inj.	inj.	-	-	-	-	Nuclear pore complex components Nup35 and Nup93	LIT/EE	16631361
23	Positive	Y49E10.14	**PIE-1**	F59B2.6	**ZIF-1**	inj.	inj.	-	-	-	-	mRNA 3'-UTR binding activity/ubiquitin-dependent protein	CePRS	
24	Positive	T27F2.1	**SKP-1**	K08B4.1	**LAG-1**	n.c.	n.c.	-	-	-	-	SNW1 (SNCSL (CBF-1, Su(H), Lag-1)W domain containing 1)/	CePRS	
25	Positive	T07E3.4	**T07E3.4**	F46A9.4	**SKR-2**	inj.	inj.	-	-	-	-	SKP1/F-Box Domain	CePRS	
26	Positive	Y39G10AR.12	**TPXL-1**	K07C11.2	**AIR-1**	inj.	inj.	-	-	-	-	Interaction between Aurora A kinase and microtubule binding protein TPX2	LIT/EE	16054030
27	Positive	Y54E5B.4	**UBC-16**	F54G8.4	**NHL-1**	n.c.	n.c.	-	-	-	-	UBE2W (ubiquitin conjugating enzyme E2 W)/TRIM2 (tripartite motif containing 2) and TRIM3 (tripartite motif containing 3)	CePRS	

Key to Abbreviations
Yes = Line successfully established and analyzed by SIMPL ELISA platform
n.c. = not cloned / cloning unsuccessful
inj. = injected, but no transgenic line could be established with expression of both Bait and Prey
CePRS = *C. elegans* Positive Reference Set
LIT/EE = Literature derived and previously confirmed by yeast two-hybrid.
Description/Homolog information from WormBase

Supplementary Figure 1

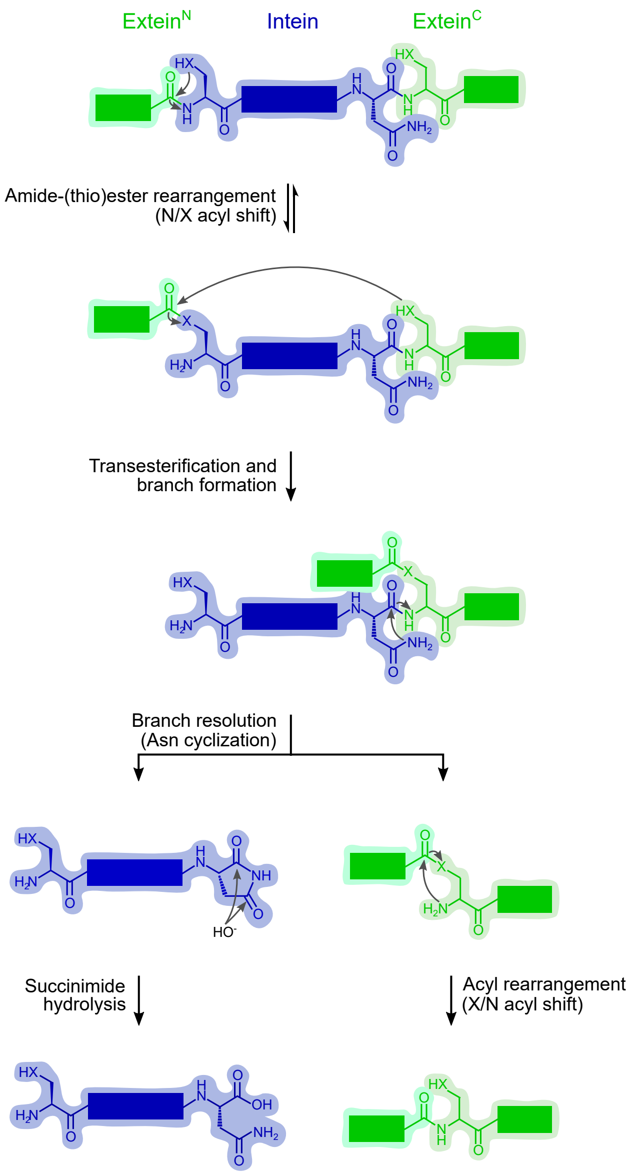

Supplementary Figure 1. Mechanism of protein splicing reaction.“X” stands for a sulfur (“S”)or oxygen (“O”) atom.

Supplementary Figure 2

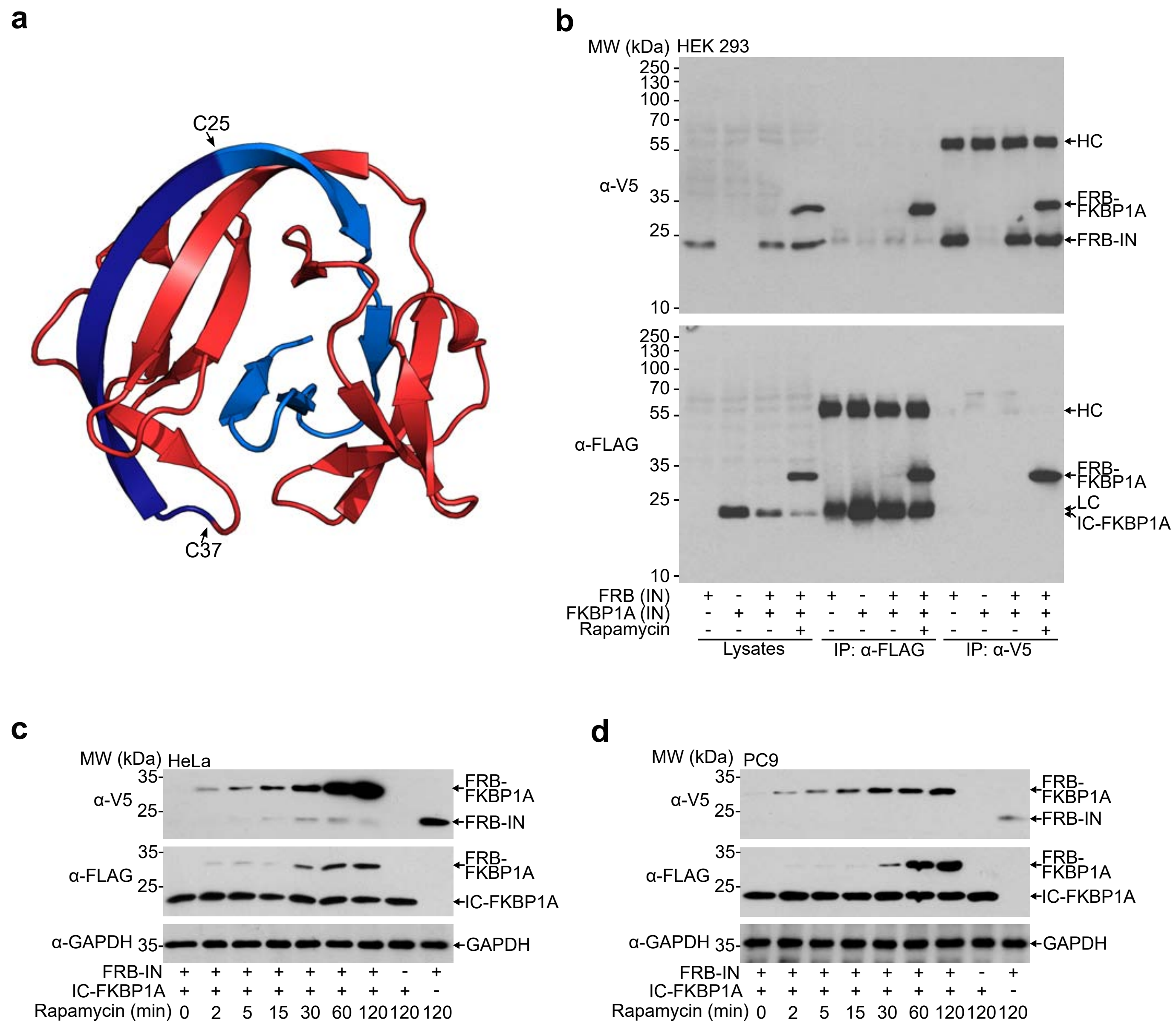

Supplementary Figure 2. Setting up the SIMPL system. **(a)** Crystal structure of GP41-1 split intein. Crystal coordinate file 6QAZ was retrieved from PDB (Beyer et al, 2019). The natural splitting site at C37 and the resplitting site (C25) used in the SIMPL system are highlighted. **(b)** Characterizing the identity of spliced protein with immunoprecipitation. Cells expressing FRB-IN, IC-FKBP1A or both were treated with rapamycin or left untreated. The proteins were immunoprecipitated with α-FLAG or α-V5 antibodies and subjected to Western analysis with indicated antibodies. HC: antibody heavy chain; LC: light chain. The blot is representative for three independent experiments. **(c-d)** Time course of rapamycin-induced FRB/FKBP interaction in HeLa (c) and PC9 (d) cells. Cells were transfected with FRB-IN and IC-FKBP1A and treated with rapamycin (100 nM) for different periods of time, as indicated, followed by Western blot analysis. The blot is representative for three independent experiments.

Supplementary Figure 3

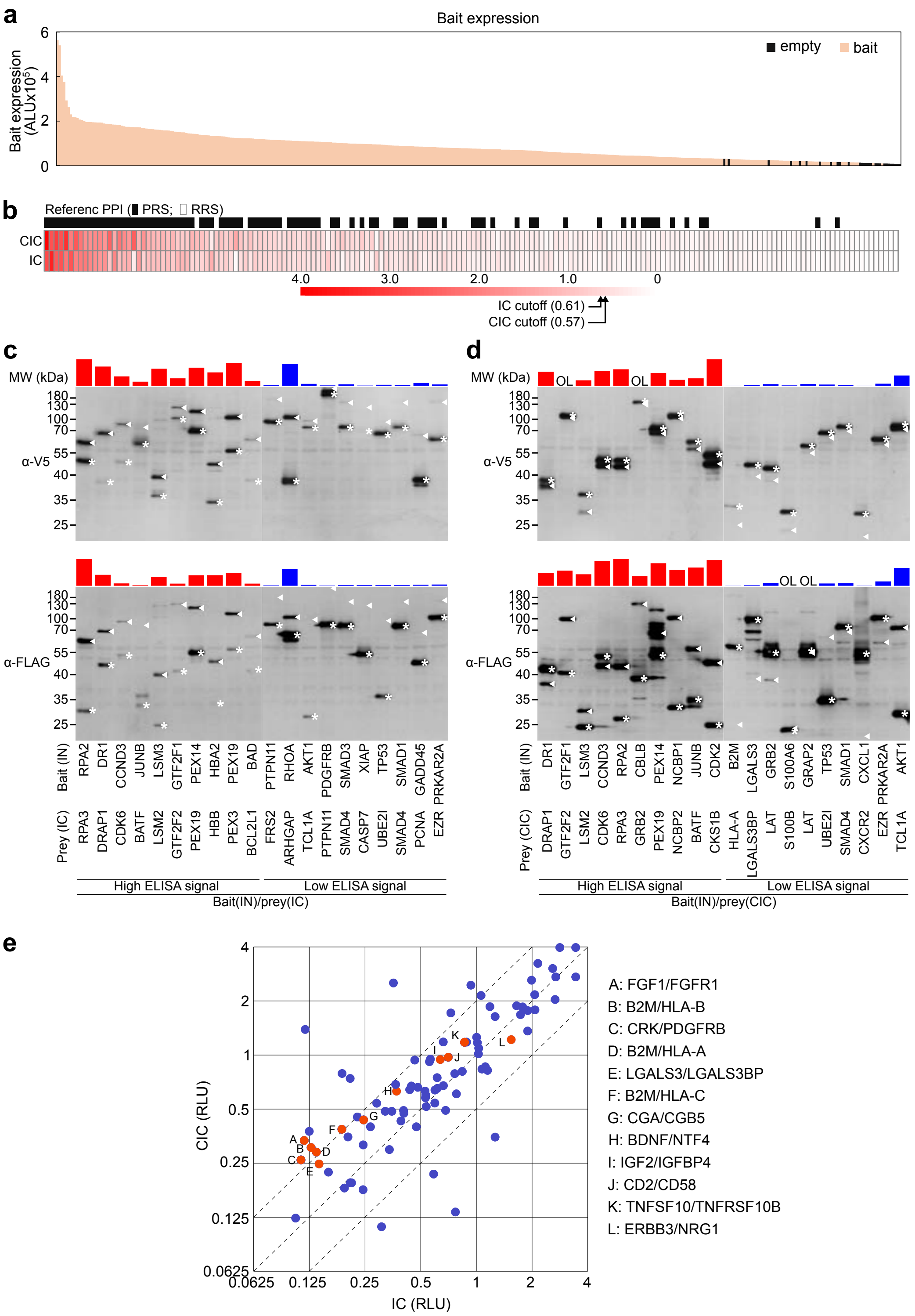

Supplementary Figure 3. Performance of ELISA coupled SIMPL evaluated with reference PPIs. **(a)** Plot of bait expression for data in Fig. 3d. Samples with mock transfection or unrelated vectors are highlighted with black. **(b)** Heatmap of splicing signal (normalized by bait signal) with prey in either IC format or CIC format. PRS pairs are symbolized with black rectangles and the RRS pairs are symbolized with white rectangles in the upper row. **(c-d)** Comparison of SIMPL ELISA and Western blot readouts. PPI pairs from the PRS, either in IN/IC (c) or IN/CIC format (d), with high signal or low signal obtained in ELISA assay, were re-tested by Western blot. Bands of parental proteins are highlighted with asterisks. Bands of spliced proteins or their predicted positions are labelled with triangles. The densities of spliced bands were quantified and are presented as bar graphs above the blots. OL indicates overlap of a spliced band and its parental bands, which prevents accurate measurement of the band. In each panel, electrophoreses were performed on two separate gels but the samples were transferred and probed on the same nitrocellulose membrane. **(e)** Comparison of the performance of IC and CIC prey formats. The SIMPL readings in (b-c) from the PRS analysis (Fig. 3d) are plotted as IC vs CIC. Prey proteins with signal peptides are highlighted in red and listed at the right side of the scatter plot. Data are presented in logarithmic scale.

Supplementary Figure 4

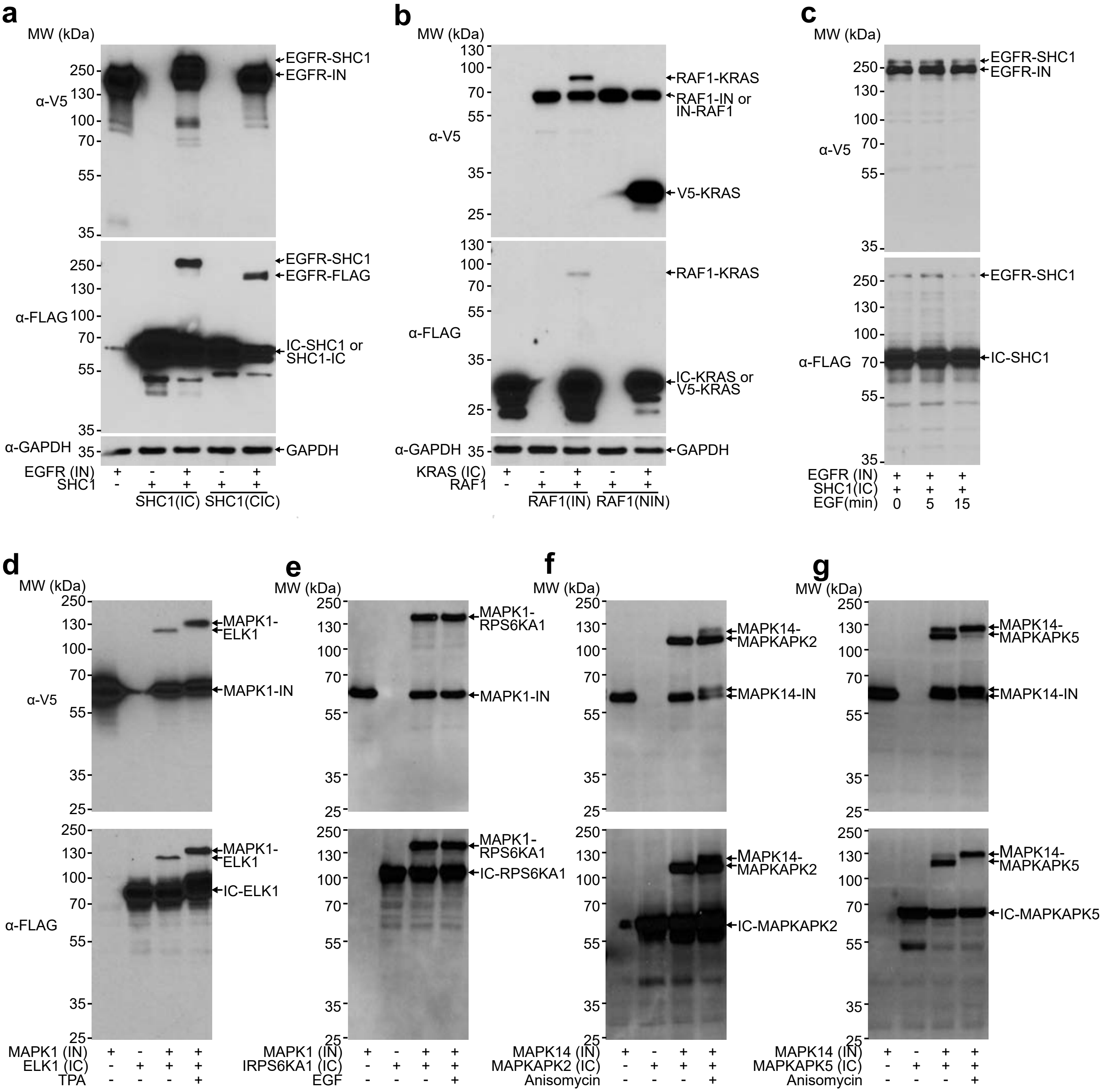

Supplementary Figure 4. Using SIMPL to study physiological PPIs. **(a)** The interaction between EGFR (in IN format) and SHC1 were detected with either SHC1 (IC) or SHC1 (CIC) formats. **(b)** KRAS (in IC format)/RAF1 interaction were detected with both RAF1-IN and NIN-RAF1 formats. Each blot in (a,b) is representative for three independent experiments. **(c)** EGFR (IN) and SHC1 (IC) were co-transfected into HEK 293 cells. After 16 hours starvation (DMEM supplemented with 0.1% FCS), the cells were stimulated with EGF (100 ng/ml) for indicated periods of time. The cells were subjected to Western blot analysis. The blot is representative for four independent experiments. **(d-g)** Kinase/substrate interactions were followed by SIMPL. The plasmids coding the indicated kinases in IN format and related substrates in IC format were transfected in HEK 293 Flp-In T-Rex cells. The expression was induced by incubation with tetracycline for 6 hrs in starvation medium. The indicated kinases are either in basal state or activated by related stimulation for 30 mins. Activation of the kinases can be judged by the mobility up-shift of substrate and spliced bands derived from phosphorylation. Each blot in (d-g) is representative for two to three independent experiments.

Supplementary Figure 5

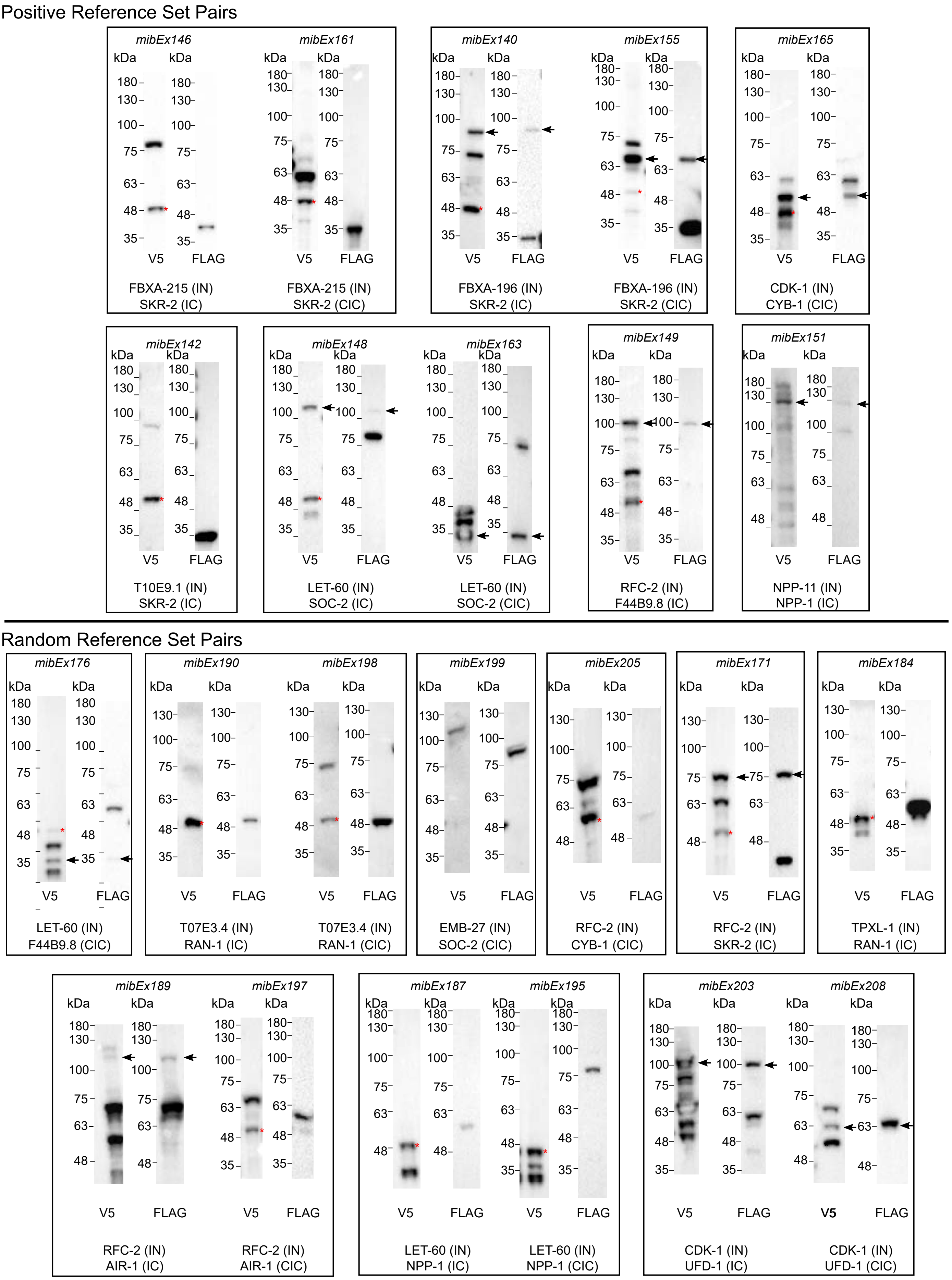

Supplementary Figure 5. Western blot screen results of *C. elegans* lines**.** Western blot results for the *C. elegans* SIMPL lines generated. Analysis of each protein pair consists of a blot using the V5 antibody to detect the Prey protein, and the FLAG antibody to detect the Bait protein. Size in kilodaltons is shown for each blot. Red asterisk indicates a non-specific V5 band, while splicing is indicated by a black arrow at the expected product size. The blot is representative for two independent experiments.
