# Supplementary material for: Split Intein-Mediated Protein Ligation for detecting protein-protein interactions and their inhibition
Source: Nat Commun. 2020 May 15;11:2440. doi: 10.1038/s41467-020-16299-1 (PMC7229206; doi:10.1038/s41467-020-16299-1)
Supplement: Supplementary file 3 — Reporting Summary [file 41467_2020_16299_MOESM3_ESM.pdf]

nature research

Corresponding author(s): Igor Stagljar

Reporting Summary

Nature Research wishes to improve the reproducibility of the work that we publish. This form provides structure for consistency and transparency in reporting. For further information on Nature Research policies, see Authors & Referees and the Editorial Policy Checklist.

Statistical parameters

When statistical analyses are reported, confirm that the following items are present in the relevant location (e.g. figure legend, table legend, main text, or Methods section).

n/a	Confirmed	
☐	☒	The exact sample size (n) for each experimental group/condition, given as a discrete number and unit of measurement
☐	☒	An indication of whether measurements were taken from distinct samples or whether the same sample was measured repeatedly
☒	☐	The statistical test(s) used AND whether they are one- or two-sided<br>*Only common tests should be described solely by name; describe more complex techniques in the Methods section.*
☒	☐	A description of all covariates tested
☒	☐	A description of any assumptions or corrections, such as tests of normality and adjustment for multiple comparisons
☒	☐	A full description of the statistics including central tendency (e.g. means) or other basic estimates (e.g. regression coefficient) AND variation (e.g. standard deviation) or associated estimates of uncertainty (e.g. confidence intervals)
☒	☐	For null hypothesis testing, the test statistic (e.g. F, t, r) with confidence intervals, effect sizes, degrees of freedom and P value noted<br>*Give P values as exact values whenever suitable.*
☒	☐	For Bayesian analysis, information on the choice of priors and Markov chain Monte Carlo settings
☒	☐	For hierarchical and complex designs, identification of the appropriate level for tests and full reporting of outcomes
☒	☐	Estimates of effect sizes (e.g. Cohen's d, Pearson's r), indicating how they were calculated
☐	☒	Clearly defined error bars<br>*State explicitly what error bars represent (e.g. SD, SE, CI)*

Our web collection on statistics for biologists may be useful.

Software and code

Policy information about availability of computer code

Data collection	ELISA data were collected with CLARIOstar.
Data analysis	Data were analyzed with Microsoft Excel Version 2002. Density of Western blot analysis was measured and calculated using ImageJ 1.8.0.

For manuscripts utilizing custom algorithms or software that are central to the research but not yet described in published literature, software must be made available to editors/reviewers upon request. We strongly encourage code deposition in a community repository (e.g. GitHub). See the Nature Research guidelines for submitting code & software for further information.

Data

Policy information about availability of data

All manuscripts must include a data availability statement. This statement should provide the following information, where applicable:

- Accession codes, unique identifiers, or web links for publicly available datasets
- A list of figures that have associated raw data
- A description of any restrictions on data availability

All data generated or analysed during this study are included in this published article (and its supplementary information files).

Field-specific reporting

Please select the best fit for your research. If you are not sure, read the appropriate sections before making your selection.

☒ Life sciences ☐ Behavioural & social sciences ☐ Ecological, evolutionary & environmental sciences

For a reference copy of the document with all sections, see nature.com/authors/policies/ReportingSummary-flat.pdf

Life sciences study design

All studies must disclose on these points even when the disclosure is negative.

Sample size	In the benchmarking analysis (ROC analysis) of the performance of SIMPL ELISA assay (Fig. 4d), we tested 88 PPIs in PRS and 88 pairs in RRS. The PRS was derived from previously descried hPRS (Nature Methods 6: 91-97) which contains 92 PPIs, of which 4 were not available in the current study. The same number of RRS pairs was chosen to match PRS with 1:1 ratio as in original evaluations of PPI detection methods.
Data exclusions	No data exclusion.
Replication	ROC analysis were performed independently three times and the data were summarized in Fig. 4e. All of them were successful and presented good consistency. Other SIMPL analyses presented in figures were performed at least 3 times and most of them were successful and showed consistent results.
Randomization	In ROC analysis, pairs in either PRS or RRS were allocated alphabetically and therefore without bias related to their biological function or biochemistry. No randomization was made in other experiments.
Blinding	In the ROC analysis, the library DNA were preformatted in a 384 well plate in an alphabetical order and aliquots were transferred to wells of cultured cells in the same format. There was no blinding in other experiments.

Reporting for specific materials, systems and methods

Materials & experimental systems

n/a	Involved in the study
☐	☒ Unique biological materials
☐	☒ Antibodies
☐	☒ Eukaryotic cell lines
☒	☐ Palaeontology
☒	☐ Animals and other organisms
☒	☐ Human research participants

Methods

n/a	Involved in the study
☒	☐ ChIP-seq
☒	☐ Flow cytometry
☒	☐ MRI-based neuroimaging

Unique biological materials

Policy information about availability of materials

Obtaining unique materials	The plasmids created in this study are available from the corresponding author upon reasonable request.

Antibodies

Antibodies used	FLAG (M2), V5, HA
Validation	FLAG (Sigma-Aldrich #F1804, lot SLBW3851): https://www.sigmaaldrich.com/catalog/product/sigma/f1804?lang=en&region=CA&gclid=Cj0KCQjw9ZzzBRCKARIsANwXaeLwCPkC8eDxDzg7csuYaBUSgVsBBGBjelv8EyTWQQYL3Rfoi27sK_YaAu5VEALw_wcB<br>V5 (Cell Signaling Technology #13202, Lot 6): https://www.cellsignal.com/products/primary-antibodies/v5-tag-d3h8q-rabbit-mab/13202<br>HA (GeneTex #GTX115044, Lot 42730): https://www.genetex.com/Product/Detail/HA-tag-antibody/GTX115044

Eukaryotic cell lines

Policy information about cell lines

Cell line source(s)	HEK 293 Flp-In T-REx, HeLa and PC9
Authentication	HEK 293 Flp-In T-REx cell line was authenticated using GenePrint 10 assay (Promega). HeLa and PC9 cells were not authenticated.
Mycoplasma contamination	HEK 293 Flp-In T-REx cell line was confirmed to be free of mycoplasma contamination. HeLa and PC9 cells were not tested.
Commonly misidentified lines (See ICLAC register)	No commonly mis-identified cell lines were used.
